# Mining the Protein Data Bank to Differentiate Error from Structural Variation in Clustered Static Structures: An Examination of HIV Protease

**DOI:** 10.3390/v4030348

**Published:** 2012-03-05

**Authors:** Balasubramanian Venkatakrishnan, Miorel-Lucian Palii, Mavis Agbandje-McKenna, Robert McKenna

**Affiliations:** Department of Biochemistry and Molecular Biology, University of Florida, Gainesville, FL 32610, USA; Email: balavenkat@ufl.edu (B.V.); mlpalii@gmail.com (M.-L.P.); mckenna@ufl.edu (M.A.-McK.)

**Keywords:** B-factor and spatial variation, data mining, HIV protease, structure superposition

## Abstract

The Protein Data Bank (PDB) contains over 71,000 structures. Extensively studied proteins have hundreds of submissions available, including mutations, different complexes, and space groups, allowing for application of data-mining algorithms to analyze an array of static structures and gain insight about a protein’s structural variation and possibly its dynamics. This investigation is a case study of HIV protease (PR) using in-house algorithms for data mining and structure superposition through generalized formulæ that account for multiple conformations and fractional occupancies. Temperature factors (*B*-factors) are compared with spatial displacement from the mean structure over the entire study set and separately over bound and ligand-free structures, to assess the significance of structural deviation in a statistical context. Space group differences are also examined.

## 1. Introduction

### 1.1. The Protein Data Bank

Established in 1971, the Protein Data Bank (PDB) has proved invaluable not only to the research community but also to students and educators [1]. The PDB has outgrown its initial purpose as a repository of the atomic coordinates of protein structures [2] and now contributes to the understanding of biological function by structural genomics and similar initiatives.

Over 71,000 structures were in the database at the time of this writing, and more are deposited weekly [3]. Considerable molecular dynamics work has been done to assess conformational changes and mobility in individual macromolecules, but looking at an entire array of static structures is an untapped approach. Extensively characterized proteins have hundreds of structures available in the PDB, including mutations, various inhibitor complexes, and different resolutions or crystallographic space groups. This provides a unique opportunity to apply data-mining algorithms to the multitude of static coordinates deposited in the PDB, obtaining a measure of reliability when deciding on the significance of a structural change, as well as possibly revealing an alternative, dynamic view of a protein.

### 1.2. HIV protease

The protein chosen as a case study to demonstrate this approach is the protease of the human immunodeficiency virus (HIV), the causative agent of acquired imunodeficiency syndrome (AIDS). The role of HIV protease (PR) in the maturation of the virus to an infective state has made it an attractive drug target. Several PR inhibitors have been used in AIDS therapy [4,5]. Highly Active Anti-Retroviral Therapy (HAART) combines multiple drugs, significantly improving prognoses [6]. However, the high mutation rate of the virus has given rise to a number of polymorphs, including drug-resistant mutants [5,7,8]. This has prompted extensive study of the mechanisms of inhibitor action and resistance in the various polymorphs. Recent investigations have looked at the structure of PR in different polymorphic forms, in both the bound and ligand-free state [8,9].

**Figure 1:**
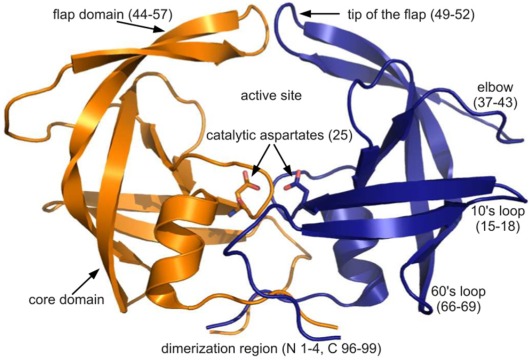
The PR dimer. Cartoon diagram of PDB ID 3hvp showing the monomers in orange and blue. Regions of PR structure are labeled, and relevant residue numbers are given in parenthesis. Rendered using PyMOL.

PR is a homodimer, as shown in figure 1. Each of the 99-residue monomers contributes a catalytic aspartate (D25) to the active site, located above the dimeric interface and enclosed by a pair of flaps [10]. The dynamic nature of the flaps has not prevented crystallographic examinations of PR, and numerous studies have worked toward elucidating the structural basis of drug action and resistance [11]. 

**Figure 2:**
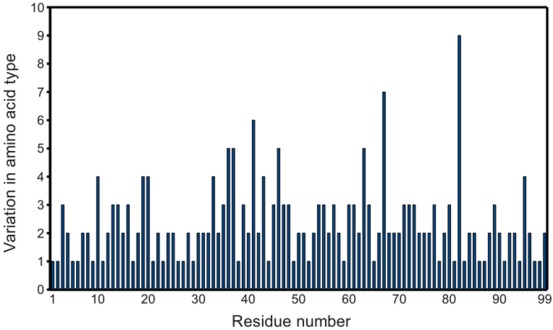
Variability of PR primary structures in the PDB. Graph considers the 811 PR monomers obtained as described in the experimental section, except PDB ID 2rkg, which contains an insertion. Non-standard residues (e.g., norleucine) are also included.

Since the first complete crystal structure of HIV-1 protease was solved [10], several hundred PR structures have been deposited in the PDB, covering significant variation in amino acid sequence, as shown in figure 2. The availability of such a substantial data set makes possible statistical probing into the properties of PR.

Presented here is an investigation using a set of in-house tools to data-mine the PDB, superpose structures, and calculate various parameters by residue or by structure. Mean temperature factors (*B*-factors) and spatial displacements were examined and correlated to resolution, ligand presence, and space group, and the obtained results were compared to current biological views. 

## 2. Results and Discussion

**Figure 3:**
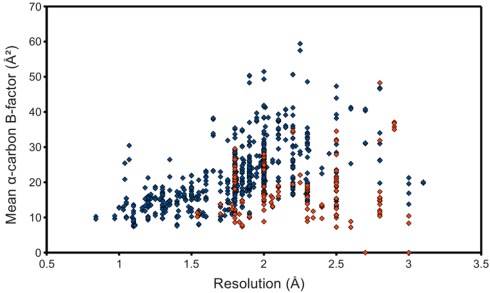
Quality of deposited PR structures. Monomers that passed the *B-*factor cut-off of 2.15 Å^2^ are marked blue, whereas those that failed are red. NMR structures, to which the concept of resolution does not apply, were not used for this plot.

The occupancy-weighted average α-carbon *B*-factor was calculated for each of the resulting chains and plotted as the ordinate of a graph using structure resolution as the abscissa, in the hope of observing an association, even if possibly a weak one. However, figure 3 shows at best a resolution-dependent upper bound for the mean C *B*-factor. The lack of a stronger association can at least partially be attributed to the surprisingly low *B*-factors reported by some structures. Many files contained atomic coordinates with *B*-factors of 2 Å^2^ and below, and several included negative *B*-factors. It was therefore necessary to remove from the study-set any structures containing *B*-factors lower than some reasonable value. This cut-off value came from a high resolution structure with reliable low *B*-factors, with no negative values. The highest resolution structure of lysozyme in the PDB at the time of this writing, PDB ID 2vb1 [14], lists no *B*-factors lower than 2.15 Å^2^, so this was selected as the cut-off. 597 HIV protease chains passed, represented in figure 3 as blue points. This filtering step noticeably improved the linearity of the relationship between resolution and C *B*-factors. 

**Figure 4:**
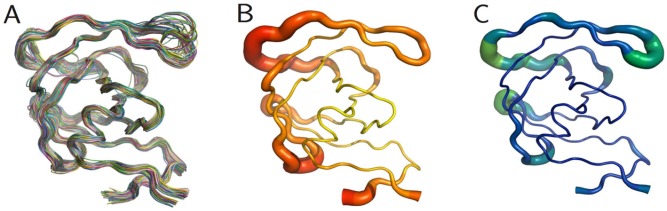
PR monomers. (**A**) Ribbon diagram of the final data set superposed by least squares. (**B**) Putty cartoon of *B-*factor variation on the mean structure, colored from low to high (yellow to red). (**C**) putty cartoon of spatial variation on the mean structure, colored from low to high (blue to green). Refer to figure 1 for definitions of PR regions. Rendered using PyMOL (DeLano, 2002)

The PR monomers composing the study-set were superposed by the least squares method. Shown in figure 4A, the ribbon diagram representation of this superposition resembles an ensemble of NMR structures, even though no NMR structures were present in the data set. Though motion cannot be inferred directly from crystallographic data, it is worth noting that the greatest variation is observed in the flap and elbow, supporting the findings of NMR and molecular dynamics studies that have described these regions as the most dynamic [18]. Interestingly, the flap region showed a greater relative thermal stability. Figure 4B, a putty cartoon based on mean Cα *B*-factors, shows much greater values in the elbow and 60’s loop than in the flap. However, when considering spatial displacement from the mean monomer (as in figure 4C), the tip of the flap joins the elbow and the 10’s and 60’s loops (defined as in figure 1) as one of the most variable regions, even though some of the range suggested by figure 4A has been averaged out. 

A possible explanation would be the existence of two distinct conformations of the enzyme: open and closed. In the latter, the presence of a ligand would enable interactions that hold the flap closed, ensuring its stability. In the former, steric clashes with symmetry-related molecules may limit flap opening and movement, or alternate conformers may be induced by amino acid variation. An analysis of crystal contacts across the various space groups mentioned in Table 1 affirms that there are several crystal contacts on the flap and elbow regions. Residues that formed crystal contacts in all the structures within each space group were used to calculate consensus contact regions within the space group. Though there are regions of contact that are specific to some space groups, the elbow and flap stretch and a number of other key contact points were common for all the space groups. 

**Table 1:** Distribution of crystal contacts by residue in representative structures from each of the space groups reported for PR. This is not a table but a figure. Author need to use Word Table tools to format table.

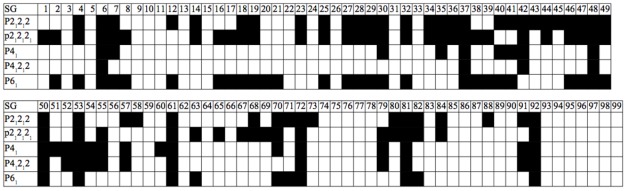

**Figure 5:**
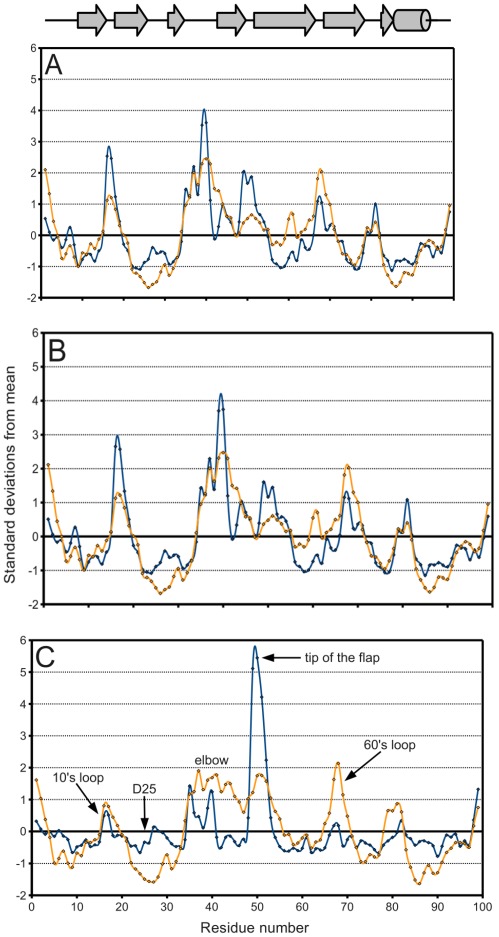
PR *B*-factor (orange) and spatial displacement (blue) variation with residue number. (**A**) Final data set, (**B**) bound monomers, (**C**) ligand-free monomers. Values were normalized for comparison purposes. Mean and standard deviation values are given in Table 2. Secondary structure elements are identified.

Figure 5A, a plot of the range of temperature factors and spatial displacement by residue, confirms the elbow, the tip of the flap, and the other loops as maxima. Overall, the *B*-factor seems to adequately fulfill its role as a *de facto* measure of spatial variation because there is high agreement in the location of the extrema of the two data series. However, the *B*-factor is not as reliable in predicting the magnitude of these extrema. Crystal contacts deduced from structures in different space groups seemingly coincide with regions of higher *B*-factors which may support the effect of crystal artifacts on the actual dynamics and *B-*factor values. The unreliability is especially apparent when separately treating ligand-bound (figure 5B) and ligand-free (figure 5C) monomers. The majority of PR structures in the PDB are bound to a ligand, so figure 5A,B do not differ significantly. 

In figure 5C on the other hand, the tip of the flap exhibits by far the greatest displacement from the mean ligand-free structure, and this value is much larger than the corresponding *B*-factor might indicate. Furthermore, the distribution of ligand-free structures is as a whole more variable spatially than that of bound ones, as described in Table 2. Spatial displacement over ligand-free structures has both a greater mean, 0.577 Å, and a greater standard deviation, 0.465 Å, than over bound structures (mean = 0.343 Å, standard deviation = 0.160 Å). The difference may be partially due to the discrepancy in sample sizes, but it nevertheless suggests the possibility of multiple PR conformations in the absence of a ligand. 

**Table 2:** PR B-factor and spatial displacement distribution. This table shows the mean spatial displacement observed in the ligand-bound Vs ligand-free PR structures. This table is a figure.

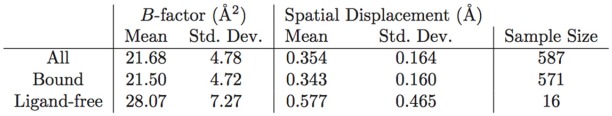

From this analysis of an entire array of structures, it is also possible to obtain an estimate of what Å value represents a significant conformational change. Referring to the statistics in Table 2, a change of 0.5 Å or below is within error range. A spatial displacement of 1.0 Å, or approximately four standard deviations from the mean of the entire study-set, is more convincing. In the distance matrix of pairwise *RMSD*s, of which a small segment is given as Table 3, several structures, namely PDB IDs 1xl2, 2fns, 2fnt, 2hs1, 2hs2, and 5upj, have *RMSD*s of 0.95 Å and above separating the monomers that compose them, supporting the finding that the two monomers adopt different conformational states when PR binds an asymmetric ligand [19]. The initial work of Prabu-Jeyabalan *et al.* [19] was on an inactivated HIV-1 PR-substrate complex, and the two monomers in the reported structure (PDB ID 1f7a) have an *RMSD* of only 0.34 Å. However, of the aforementioned structures, only PDB IDs 2fns and 2fnt have peptide ligands whereas the rest are bound to non-peptides, and PDB ID 5upj is an HIV-2 PR. The observation may therefore be conjectured to hold generally for HIV proteases and asymmetric ligands.

**Table 3:** Representative table of the pairwise RMSD distance (Å) matrix of the 587 monomers in the study set. Rows and columns are labeled with the PDB ID and chain identifier. This is a figure.

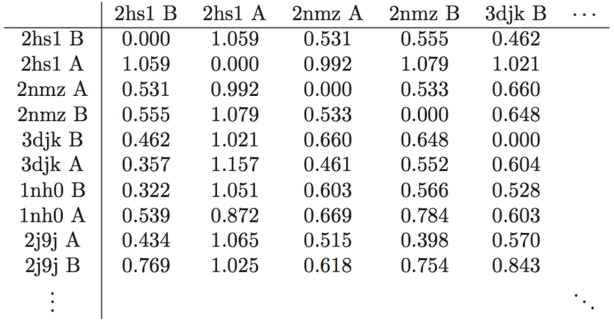

**Figure 6:**
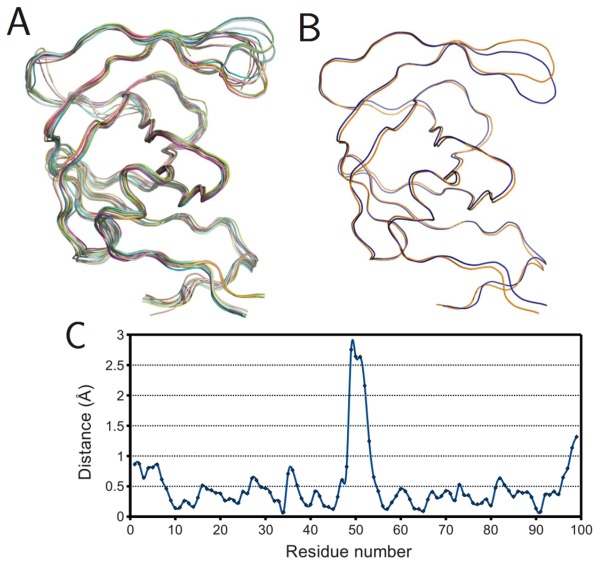
Ligand effects on PR monomer structure. (A) Superposition of ligand-free monomers; (B) superposition of mean ligand-free (orange) and bound (blue) monomers; (C) plot of spatial difference Vs residue number for mean ligand-free and bound monomers. Ribbon diagrams rendered using PyMOL.

To further understand the effects of ligand binding on PR structure, monomers were superposed within the bound and ligand-free subsets. Figure 6A shows the result for the ligand-free monomers. Surprisingly, not all structures had flaps in the “semi-open” or open conformations. Several exhibited the closed flap conformation, though closer examination revealed these to belong to covalently-bonded PR dimers (PDB IDs 1g6l and 1lv1) that were split into monomers by removing the bridge of connecting residues. This also explains why these structures differ noticeably at the C-terminus from the other ligand-free structures. The superposition of the mean bound and mean ligand-free monomers rendered as a ribbon diagram in figure 6B, shows a prominent difference in the tip of the flap but little variation elsewhere. Figure 6C gives the same information as a plot of spatial difference by residue, and the spike corresponding to the tip of the flap is unmistakable. However, the maximal distance, 2.75 Å, is smaller than the actual deviation between the open and closed conformations, because the mean ligand-free structure is closer to the semi-open state due to averaging.

**Figure 7:**
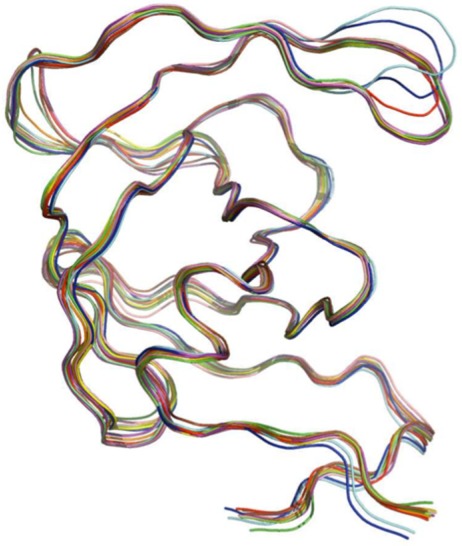
Superposition of mean PR monomer structures for the space groups: P2_1_ (orange), C2 (red), P2_1_2_1_2 (chartreuse), P2_1_2_1_2_1_ (yellow), I222 (purple), P4_1_ (cyan), P4_3_ (lime green), P4_1_2_1_2 (blue), P4_3_2_1_2 (magenta), I4_1_22 (salmon), P6_1_ (olive), P6_5_ (brown), P6_1_22 (pink) and I2_1_3 (green). Ribbon diagram rendered using PyMOL. Table 3 describes the distribution of space groups in the final data set.

Monomers were also organized on the basis of crystallographic space group and superposed to obtain mean monomers. Most of the representative monomers were in the closed conformation, as shown in figure 7. Exceptions were the monomers corresponding to the C2, P4_1_2_1_2, and P4_1_ space groups. Interestingly, all structures that crystallized in the C2 space group were bound to a ligand, as listed in Table 4. This surprising observation may be due to the fact that all C2 structures except PDB ID 1ztz were of HIV-2 PR and solved during the same study. The deviation of the P4_1_2_1_2 space group is accounted for by noting that all its monomers are ligand-free, except one (PDB ID 3bc4 [20]) whose flaps are prevented from closing by two non-peptide inhibitors that pack the active site, acting as a wedge. Finally, the P4_1_ space group has an almost equal distribution of bound and ligand-free monomers but differs the most from the closed conformation, which would be expected of a predominantly ligand-free space group. This may be because most of the P4_1_ structures have mutations at residues 82 and 84, which are essential to ligand binding and structural stability in the active site [21]. 

**Table 4:** Distribution of PR structures by space group. In the strictest sense, the distribution should be further subdivided because not all structures belonging to the same space group have isomorphous unit cells.

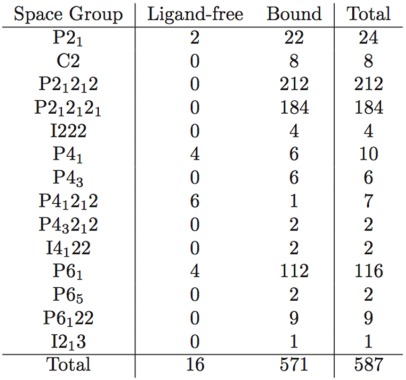

## 3. Experimental Section

### 3.1. Data-mining the Protein Data Bank

A list of relevant PDB IDs was obtained by querying the PDB for structures matching the keywords “HIV protease.” The same 174 hits were returned regardless of whether the query was executed programmatically or through the PDB’s web interface. Expanding the search parameters to include “human immunodeficiency virus protease” increased the number of results to 405. However, a review of the literature revealed that the true number of relevant structures in the PDB is greater still. Using the aforementioned search parameters, a home-grown script that does not limit itself to phrases explicitly declared as keywords found an additional 34 PDB IDs. Several of the previously-missed files were in fact PR structures that had managed to evade normal search mechanisms by having only general keywords, such as hydrolase.

### 3.2. Refining the search results

Of the 439 PDB IDs obtained, many corresponded not to PR, but to other proteins, including but not limited to integrase, reverse transcriptase, and proteases of the simian immunodeficiency viruses and feline immunodeficiency viruses (SIV and FIV, respectively). Therefore, a screening tool was implemented to omit structures containing no chains with primary structures within a specified edit distance [12] of several reference PR sequences from both HIV-1 and HIV-2. The two variants exhibit very similar overall structure despite having only about 50% sequence identity [13]. The results of the screen still had to be checked manually; SIV protease structures passed this test due to high sequence homology with PR. Conversely, covalently bonded PR dimers failed due to high edit distances; one of the monomers as well as any connecting residues had to be deleted to match the reference sequences. Hence, SIV proteases were removed, and tethered HIV proteases were readded. Despite the limitations, developing tools to facilitate the tedious task of selecting search results is an essential step towards the ultimate goal of having complete data mining packages to take advantage of the ever-increasing volume of information contained in the PDB.

### 3.3. Quality control

The 368 structure files remaining after the previous step included several structures from NMR experiments and were split into 811 PR monomers. In the case of the covalently bonded PR dimers, this involved stripping any connecting residues. NMR structures were excluded from the study-set. The final data-set can be found in the supplementary data section.

### 3.4. Structure superposition

The problem of superposing two sets of three-dimensional coordinates (atomic or not) reduces to identifying the rotation and translation that minimize some error function, usually the root-mean-square deviation (*RMSD*) between the coordinate sets in question: 


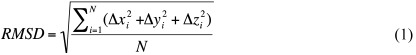


 Because *N* is just a constant, and the square root is a strictly increasing function, this is equivalent to the least squares criterion. In this case, the optimal translation can be calculated independent of rotation and is well known as the vector separating the centroids of the two coordinate sets [15].

Several methods are known for computing the optimal rotation. The most popular seems to be that of Kabsch [16], but this approach represents rotations as 3 x 3 matrices and can give rise to rotoinversions. For this investigation, the method of Horn [17] was chosen because it circumvents this problem by instead representing rotations as unit quaternions. Letting (*x_Ai_, y_Ai_, z_Ai_*) denote the displacement of the *i*th point in set *A* from its centroid, the optimal rotation becomes the eigenvector corresponding to the most positive eigenvalue of the symmetric 4 X 4 matrix: 


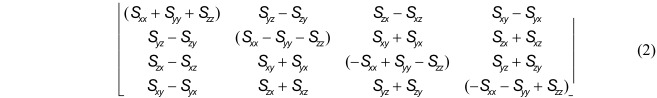


where *S_xx_*= Σ*_i_ x_Ai_x_Bi_*, *S_xy_*= Σ*_i_ x_Ai_y_Bi_*, and so on.

Applying this algorithm to the atomic coordinates of protein structures involves accounting for fractional occupancies and multiple conformations. This amounts to using the occupancy-weighted centroids for the translation step and generalizing *x_Ai_* to the occupancy-weighted average of its conformations *j*, 


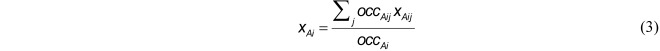


and similarly for *y* and *z*, where *occ_Aij_* denotes the occupancy of the *j*th conformation and *occ_Ai = _*Σ*_j_occ_Aij_* is the total occupancy corresponding to (*x_Ai_, y_Ai_, z_Ai_)* . However, the *S* summations are also occupancy-weighted, which factors out the denominators in (3), and S*_xy_*, for example, becomes 


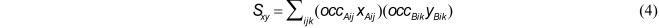


In the simple case of a single conformation with full occupancy, the formula reduces to that of Horn [17].

Unfortunately, a closed-form recipe for the superposition of multiple structures does not exist. A first approach might be to superpose all structures onto the same reference structure, but the results may be erroneous if the reference is poorly chosen. A possible improvement would be to superpose each structure onto the mean of those already considered, but even this strategy is vulnerable to the effects of an arbitrary order of superposition because structures considered earlier are inherently attributed more importance. To alleviate this problem, the 587 monomers to be analyzed were sorted from highest to lowest resolution, with ties being broken in favor of the structure with the lowest mean C^α^ *B*-factor. Equal treatment of all structures would be ideal, but preferring “better” monomers is acceptable.

## 4. Conclusions

Analysis of a static array of PDB structures to gain further insight about a protein has great potential as a method to deduce a statistical bar for structural variation, as demonstrated by this PR case study. While there are other algorithms to data-mine the PDB, it is clear from this study that quality control is required before using a data-set for analysis. There also exist algorithms to superpose structures, but to our knowledge, this is the first method that also occupancy-weighs available conformations for the superposition. The algorithms described here were used to data-mine the PDB, filter search results, perform quality control, and superpose structures. This made possible a comparison of *B*-factors and spatial variation over the entire study-set of PR monomers, the bound and ligand-free subsets, and the different represented space groups. Examination of the resulting distributions is an alternative way of identifying a protein’s most variable regions and qualifying spatial displacement as significant or within the range of error. 

However, such an approach to protein study is made more difficult by the many different practices of PDB depositors even within the limits of a file format with a detailed specification. Choice of title, choice of keywords, numbering of residues, and organization into models and chains are often overlooked. This is unnoticeable to a human user, but it makes selection of the study-set the most complex and error-prone step of a data-mining endeavor. Additionally, many structures abuse *B*-factors, occupancies, and other parameters, or assign them special meaningless values not specified by the PDB file format. Therefore quality controls must be implemented to exclude from such investigations any structures with statistics that might bias results. For data-mining investigations to be successful, a paradigm shift will be required of depositors to the PDB: to stop treating the painstaking process of preparing a structure for submission as an unnecessary complication and see the PDB itself not just as a collection of coordinates, but as a tool that could shed light on many of the questions of structural biology.

## Acknowledgments

This project could not have happened without the work of all those who have deposited structures to the PDB and those who continue to maintain a smoothly-running database. 

## Conflict of Interest

The authors declare no conflict of interest.
